# Long-Term Adverse Effects and Survival in Patients with Myasthenia Gravis Treated with Azathioprine: A Retrospective Cohort

**DOI:** 10.3390/jcm14113945

**Published:** 2025-06-03

**Authors:** Pedro J. Modrego

**Affiliations:** Neurology Department, Miguel Servet University Hospital, 50012 Zaragoza, Spain; pmpjmp@gmail.com

**Keywords:** myasthenia gravis, azathioprine, survival, adverse events

## Abstract

**Objective:** The objective of this retrospective cohort is to analyze survival and other outcomes in patients with myasthenia gravis treated with azathioprine in comparison to the standard treatment based on pyridostigmine and corticosteroids. **Methods:** A retrospective cohort of 90 patients with myasthenia gravis were followed up on for a mean period of 103.8 months. Survival/mortality was compared between patients receiving azathioprine and those on standard treatment with pyridostigmine and prednisone. Survival analysis was performed with the method of Kaplan–Meier and the Cox proportional hazards model. The long-term side-effects were also reported. **Results:** The patients on azathioprine had a longer survival according to the unadjusted log-rank test. However, in the multivariate analysis, age at baseline was the only predictor of any cause mortality (HR: 1.12; 95% CI: 1.06–1.19), but not the use of azathioprine (HR: 0.30; 95% CI: 0.10–1.43). Some malignancies appeared in patients treated for more than 10 years. Hematological abnormalities such as leucopenia, anemia, and pancytopenia occurred in four patients and malignancies in three. **Conclusions:** The use of azathioprine in MG did not result in longer survival compared to standard treatment. Some hematological alterations and malignancies may appear over time in patients receiving azathioprine.

## 1. Introduction

Myasthenia gravis (MG) is a rare condition that occurs worldwide, may appear at any age, and causes fatigability because of antibodies acting at the neuromuscular junction by blocking the motor plaque. The activation of the complement system also has an important role in the pathophysiology of MG [1]. Treatment is based on cholinesterase inhibitors and corticosteroids at the lowest possible doses [2] as first line drugs. Many patients require the use of concomitant immunosuppressant drugs to achieve remission or a minimally symptomatic state and need to decrease their dose of corticosteroids or reach withdrawal if possible. Azathioprine (AZTPA) is better tolerated than other immunosuppressive drugs, and hence is the most frequently recommended [3,4]; alternatively, other drugs can be offered such as cyclosporine, mycophenolate mofetil [4], methotrexate [5], and tacrolimus [6].

The evidence of the efficacy of AZTPA is based on several small randomized clinical trials [7,8,9,10], cohorts [11], and case series. However, serious adverse events may occur in some patients. In a series of 138 patients with MG, 53% of those treated with azathioprine developed adverse effects after a mean follow-up of 4.5 years [12].

With regard to the association between myasthenia gravis and malignancies, the information given in cohort studies is controversial. On the one hand, the risk of malignancies was evaluated in a meta-analysis of 1650 patients treated with azathioprine and 2481 not treated with azathioprine, and the risk was not significantly elevated [13]. However, in a registry-based study in Denmark, azathioprine increased the risk of non-melanoma skin cancer after 10 years of follow-up [14].

Data on survival in MG have been reported rarely; in a French cohort the myasthenic patients were at nearly twice the risk of dying compared with matched controls [15]. No data exist on whether the use of AZT may decrease mortality.

The purpose of this study was to determine the survival rate in patients on AZTPA compared to those on standard treatment, namely pyridostigmine plus prednisone; to compare the rate of exacerbations; and to determine the number and type of serious adverse events as well.

## 2. Methods

A cohort of consecutive patients with seropositive MG was recruited in the outpatient clinics of a tertiary centre. Usually, the patients were referred by family physicians, urgentists, and other neurologists. Diagnosis was based on symptoms (fatigability and weakness of the muscles) that worsened with repetitive exercise and responded to cholinesterase inhibitors (in the Tensilon Test and/or in maintenance therapy), the presence of specific antibodies in the serum (anti-AChR, anti-Musk LRP4), and the decrement response of compound muscle action potential after repetitive stimulation in the electromyography. Repetitive stimulation was usually conducted in the facial muscles with retroauricular stimulation. Seronegative MG patients (5 cases) were not included in this study. All patients were diagnosed by neurologists of the hospital facilities and confirmed in a neuromuscular diseases unit, as well as undergoing treatment and follow-up.

Classification by disease form at the onset of clinical presentation was performed according to the myasthenia gravis foundation of America.

The functional status at baseline was measured with the 8-item myasthenia gravis activities of daily living (MG-ADL), with a possible score ranging from 0 to 28 points [16]. The score was obtained in the first visit to the hospital.

After diagnosis and treatment, the patients were revised as many times as needed, and every 6 months in stable patients. Cell blood counts were performed monthly in the first 6 months, and after that, every 6 months. Pyridostigmine was the usual drug to begin with, followed by the addition of prednisone if needed, with increasing doses and posterior tapering. If no improvement was achieved, then we additionally used an immunosuppressant. AZTPA was the most common disease-modifying drug in our hospital because it was better tolerated than other immunosuppressive drugs. In refractory cases and exacerbations, we used intravenous immunoglobulins, we seldom used plasmapheresis, and we used rituximab from time to time.

The dose of AZTPA was calculated according to the weight and levels of Thiopurine-Metyl-Transferase in the blood, and it ranged from 50 to 200 mg daily.

The data collection dated back to patients seen from September 2006 to September 2024. Outcomes were retrieved retrospectively from the clinical records. The protocol was approved in February 2024. Therefore, the design of the study was a retrospective cohort. Mortality of any cause and relevant side-effects were recorded throughout the study. The number of exacerbations requiring an increase in the corticosteroid dose and/or intravenous immunoglobulins was also recorded, and complications were recorded as well.

Information on the cause of death was retrieved from hospital-based or sanitary area medical records.

The design of this cohort followed the STROBE guidelines for cohort studies (https://www.strobe-statement.org/checklists/ (accessed on 29 May 2025)).

Statistical analysis: Statistical analysis included descriptive parameters such as the means, SDs, and ranges. Comparison of means was performed by means of a *t*-test, and a X^2^ test was used for proportion comparison. The normality of quantitative variables was checked with the Kolmogorov–Smirnov test. Survival analysis was performed by means of the Kaplan–Meier method to compare survival in patients receiving AZTPA versus those on standard treatment. Cox proportional hazards model regression, which allowed us to adjust the effect of treatment for potential confounders such as age, sex, and form of the disease, was performed to adjust the model. Calculations were made with the MedCalc software Bv, version 19.1.5, Ostend, Belgium.

## 3. Results

The cohort initially included 92 seropositive patients: 3 were anti-Musk positive, and the rest were positive for anti-AchR antibodies. Two female patients with ocular myasthenia were lost to follow-up. Therefore, the data of 90 patients were available for analysis.

AZTPA was given initially to 38 patients with insufficient control of their symptoms with cholinesterase inhibitors and prednisone, but 7 patients quit the drug because of side-effects and continued with the standard treatment. Two patients who did not tolerate AZTPA are currently on cyclosporine and mycophenolate mofetil, respectively. Tacrolimus was tested in another three patients not tolerating AZTPA, but they quit the drug due to side-effects. The same occurred in two patients who took cyclosporine, with no additional immunosuppressants. Therefore, 31 patients on AZTPA were followed up on along with 59 not receiving AZTPA. In Table 1, the demographic data and follow-up periods are given. There were 50 women and 42 men.

The mean follow-up period was 103.8 months (range: 18–240 months) for the full cohort. In Table 2, the number of patients by each degree of the disease are given.

### Vaccination Status Against COVID-19 and Seasonal Flu Data

A total of 66 patients were vaccinated against seasonal flu yearly; 63 patients were vaccinated against COVID-19; and 6 did not accept any of the vaccines. Two patients fully vaccinated with an RNA-based COVID vaccine developed a COVID-19 infection in 2021 and 2022, respectively; one of them was on AZTPA, but both overcame the infection.

The mean MG-ADL baseline score for patients receiving AZTPA was 12. A total of 6 points (SD: 6.8) were scored versus 8.7 (SD: 5.9) in patients receiving the standard treatment; *t*-test: 2.81; *p* = 0.006. The mean age of patients on AZTP was 71.16 (SD: 13.34) and the mean age of those not receiving AZTP was 76.66 years; SD: 13.49; t = 1.61; *p* = 0.07.

The use of AZTPA resulted in fewer admissions to hospital and less rescue medication in fifty percent of the patients (16 patients), but the clinical course was unchanged in the rest of the treated patients. Corticosteroids were withdrawn in 14 patients, and in the rest, lower doses of corticosteroids were needed. The mean number of exacerbations was 6 (SD: 3.2) in patients under AZTPA compared to 9 (SD: 4.4) in patients under standard treatment; t = 3.31; *p* = 0.001. There were less hospital admissions due to exacerbations in patients receiving AZTPA (5 patients) than in the other group (11 patients), although the difference was not significant. Intravenous immunoglobulins was the preferred option of treatment in patients needing hospital admission.

**Figure 1:**
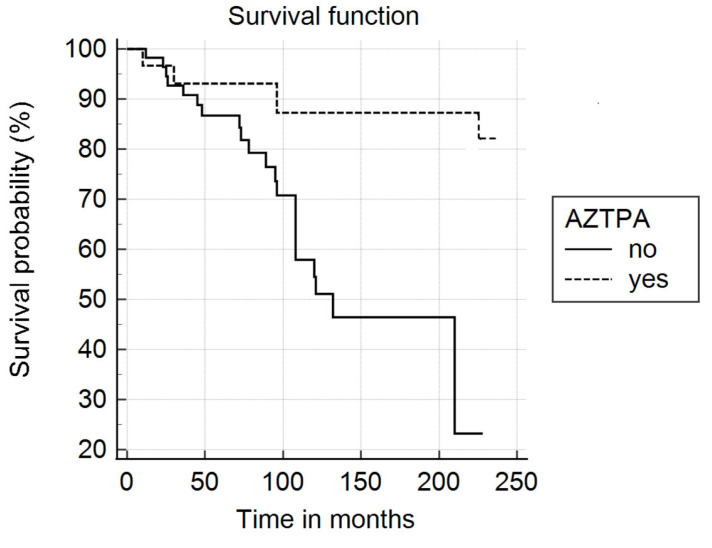
Survival function in patients on AZTPA compared to the standard treatment.

At the end of the follow-up, 65 patients were alive, and 25 had died. In the group receiving AZTPA, 5 patients died, compared to 20 in the group not receiving AZTPA. Figure 1 shows the survival curves for patients receiving AZTPA (31 patients) and those not receiving immunosuppressive drugs (59 patients).

The mean follow-up period for the azathioprine group was 105.48 months, and for the full cohort it was 103.8 months. At first glance, those receiving AZTPA had a longer survival period, with a log-rank test of 5.49; *p* = 0.018. However, when the effect was adjusted for age and disease form, the difference disappeared, and the Hazard ratio was not significant (HR: 0.38; 95% CI: 0.10–1.43). Age at baseline was a predictor of mortality (HR: 1.12; 95% CI: 1.06–1.19) and the disease form was as well (HR: 1.25; 95% CI: 1.07–1.56), see Table 3.

The mean age at baseline was 71.23 (SD: 13.5) in the patients who were alive, but it was 84 years (SD: 8.7) in those who had died after the follow-up; t = 4.29; *p* = 0.0001. Only one death was attributed to myasthenia through respiratory failure, but MG had some influence in two deaths.

With regard to the total adverse events associated with AZTPA, these happened in seventeen patients out of thirty-eight (44.7%) throughout the follow-up; in the first 3 months, one patient developed an allergic reaction, three had gastrointestinal disturbances, and another one had pancytopenia in the blood after 6 weeks. Liver enzyme elevation was seen in two patients. Withdrawal of AZTPA reversed the symptoms and normalized the blood cell count. The patients on AZTPA who withdrew from taking the drug earlier than 6 months in were included in the group of non-AZTPA-receivers for survival analysis purposes. The drug was not retested in case of withdrawal for side-effects.

During the follow-up, we encountered the following adverse events in patients kept on AZTPA: one case of pancytopenia after 15 years of treatment, one case with anemia after a 4-month period, and two cases of both anemia and thrombocytopenia after 4 and 8 years, respectively, with normalization after the withdrawal of the drug. Malignancies were seen in three patients during a follow-up for longer than 10 years: lung adenocarcinoma, Kaposi’s sarcoma, and squamous cell lung cancer. In the group of patients not receiving azathioprine, there were two cases of cancer: one patient with gastric cancer, and another one with lung cancer. Kaposi’s sarcoma was cured with withdrawal of AZTPA and the appropriate oncologic treatment.

The causes of death in the patients on AZTPA were as follows: pneumonia with sepsis in two patients, urinary infection with sepsis in one patient, respiratory insufficiency in one patient, and cardiac failure in another patient. In the group not receiving AZTPA, two cases of urinary infection with sepsis occurred; heart failure occurred in three patients, respiratory infection in eleven patients, respiratory insufficiency in two patients, and multiorgan failure in other two patients.

## 4. Discussion

In the era of new biological drugs for MG such as complement-inhibitor monoclonal antibodies (eculizumab, ravulizumab, zilucoplan) and Fc-Rn antagonists (efgartigimod and rozanolixizumab), which are already approved in Europe and in the US [17], and others which are in development, such as baclizumab, inebilizumab, and nipocalimab, azathioprine has still an important role as a disease modifier allowing clinicians to use lower doses of corticosteroids. AZTPA is cheap and well tolerated, but its effect is not immediate, and several months are needed to improve the symptoms. In addition, the benefits of AZTPA are well known worldwide, and therefore, many clinicians use it when cholinesterase inhibitors and corticosteroids are not effective. The newer drugs have a quicker effect initiation and more comfortable dosing, but they have only been compared with placebo in clinical trials, and so far they are especially indicated in refractory cases of MG. Nevertheless, these drugs look promising, and it is likely that the paradigm of treatment may change in the near future, with the increased use of high-efficacy drugs in earlier phases of the disease. To date, we do not have many studies focused on the long-term side-effects and mortality caused by AZTPA. The global mortality in this cohort of MG patients was 26.6%, which was not different from that found in a large Swedish nationwide registry of 4559 patients, which was 24.5% in 10 years [18]. In a Serbian study, the mortality rate at 30 years was 20%, with male sex and age being predictors of mortality [19]. However, a mortality rate of 43% was found after 20 years of follow-up in Denmark [20]. In the US, the age-adjusted mortality rate increased from 6.21 per million population to 9.51 from 1999 to 2022; the increase was especially higher in older and male patients [21].

Azathioprine is one of the main disease-modifying drugs for myasthenia gravis, and it is used alone or in combination with corticosteroids to achieve remission or a minimally symptomatic state. In general, this treatment is well tolerated, but adverse events may occur sooner or later throughout the disease duration. In this cohort, adverse events occurred in 44.7% of cases, which was higher than thought. In a French retrospective cohort of 138 patients with MG, azathioprine caused side-effects in 53% of patients (in 12% they were severe), which was higher than with corticosteroids (23%) or mycophenolate (15%), with a mortality rate of 5.1% for a mean follow-up of 4.5 years [21]. In another cohort of 104 patients treated with AZTPA, side-effects were seen in 35% of cases [22]. Although the risk of malignancies was not elevated in patients receiving AZTPA according to a meta-analysis [13], in a registry-based study in Denmark, azathioprine increased the risk of non-melanoma skin cancer after 10 years of follow-up [14]. In a series of 117 patients with a mean follow up of 10.48 years, 10 patients (8.5%) developed hematological complications (leukopenia, pancytopenia, thrombocytopenia, anemia plus leukopenia) [23]. In a study with 42 patients treated with AZTPA for a mean length of 6.1 years, non-melanoma skin cancer occurred in 3 (7.1%) patients [24]. In the cohort of Hohlfeld et al., five (4.8%) patients treated with AZTPA developed some malignancy, but only in a case with lymphoma was there a causal relationship [22].

AZTPA use is controversial, as MG is associated with a 39% increase in infection risk compared to controls. With regard to infections, we had a few patients with infection, but the link was determined according to the data of a Canadian population-based cohort [25].

In a cohort of 174 patients with a median follow-up of 5 years, mortality was higher in late-onset cases, and two malignancies and two infections were associated with death [26]. In this series, mortality was associated with older age and disease degree, but not with the use of azathioprine.

With regard to the effectiveness of the different used drugs in myasthenia gravis, there are no direct comparative trials, and it is difficult to demonstrate the superiority of any drugs over the others. In a retrospective Chinese cohort of 1064 patients [27], those treated with rituximab had fewer relapses than classic immunosuppressants like tacrolimus, mycophenolate mofetil, and azathioprine. The combination of tacrolimus plus corticosteroids and the combination of mycophenolate mofetil plus corticosteroids reduced the risk of relapse in comparison with the combination of azathioprine plus corticosteroids. In a small cohort, AZTPA in combination with pyridostigmine and corticosteroids decreased the number of relapses compared with the combination of pyridostigmine and corticosteroids. The use of AZTPA allowed for the dose of both pyridostigmine and corticosteroids to be decreased [28]. In a North American prospective cohort of 167 enrolled patients, with 78 included in the analysis, 47 received mycophenolate mofetil and 78 received azathioprine. There was no difference in the clinical outcomes between both groups, but AZTPA caused more potentially serious effects than mycophenolate across the follow-up of 20–25 months, which was relatively short compared to other cohorts [29]. The advantage of AZTPA over mycophenolate mofetil is the lack of teratogenicity.

The clinical outcomes of this cohort were similar to those of referenced cohorts. Our cohort had the strength of being a cohort with a longer follow-up period than other previous studies, but it was not large enough to draw definitive conclusions on survival/mortality. A non-negligible proportion of cases may develop serious side-effects with the use of AZTPA. We must remain vigilant and monitor for hematologic adverse events and malignancies, because an early detection may result in the resolution of these complications. Although the use of AZTPA had no influence on survival, it was of benefit for decreasing exacerbations and sparing corticosteroids.

There were some shortcomings in this cohort study that might influence the generalizability of the results. In this cohort, elderly patients were overrepresented. Treatment was chosen in relation with the clinical function of the patients; the patients with a worse function had a greater likelihood of receiving AZTPA than those with a better function. To draw definitive conclusions on mortality, it would be necessary to study this in larger cohorts.

The newer drugs for MG already on the market have attracted clinicians’ attention because of their promising results in clinical trials compared to placebo [17]. Notwithstanding the potential of these drugs, AZTPA with the lowest dose of corticosteroids remains a first line treatment in many countries and guidelines for treatment [30,31].

## 5. Conclusions

The use of AZTPA in MG is beneficial in patients with myasthenia gravis, but some side-effects are frequent, and potential serious side-effects may occur over the time. However, it does not seem to decrease mortality by myasthenia compared to the standard treatment.

## Figures and Tables

**Table 1:** Demographic variables.

Number of patients: 90
Mean age at baseline: 74.2 y; SD = 13.4 y; range: 32–95 y
Median age: 72 years
Sex: 48 women, 42 men
Clinical form: Ocular in 20 patients, generalized in 70
Thymoma found in three patients; two operated on
Thymectomy without thymoma found in one case
Patients on azathioprine: 31

Mean follow-up for the cohort: 103.87 months (8.6 years); SD: 51; range: 18–240 months
Median follow-up: 96 months

Mean follow-up for patients on AZTP: 105.4 months; SD: 48.7; range: 18–240 months
Median follow-up: 94 months

**Table 2:** Number of patients by degree of the disease.

Grade I. Any ocular muscle weakness; may have weakness of eye closure. All other muscle strength is normal: 20 patients
Grade IIa. Predominantly affecting limb, axial muscles, or both. May also have lesser involvement of oropharyngeal muscles: 22 patients
Grade IIb. Predominantly affecting oropharyngeal or respiratory muscles, or both. May also have lesser or equal involvement of limb or axial muscles, or both: 19 patients
Grade IIIa. Predominantly affecting limb or axial muscles, or both. May also have lesser involvement of oropharyngeal muscles: 8 patients
Grade IIIb. Predominantly affecting oropharyngeal or respiratory muscles, or both. May also have lesser or equal involvement of limb or axial muscles, or both: 6 patients
Grade IVa. Predominantly affecting limb or axial muscles, or both. May also have lesser involvement of oropharyngeal muscles: 6 patients
Grade IVb. Predominantly affecting oropharyngeal or respiratory muscles, or both. May also have lesser or equal involvement of limb or axial muscles, or both: 8 patients
Grade V. Defined as intubation, with or without mechanical ventilation, except when employed during routine postoperative management. The use of a feeding tube without intubation places the patient in class IVb: 1 patient

**Table 3:** Cox regression analysis with Hazard ratios and 95% CIs.

Predictor	Hazard Ratio	95% CI	Significance
Azathioprine	0.3897	0.10–11.43	0.156
Age	1.126	1.06–1.191	<0.0001
Clinical form	1.259	1.07–1.56	0.033
Sex	0.54	0.23–1.26	0.15

## Data Availability

The raw data supporting the conclusions of this article will be made available by the authors on request.

## References

[1] Howard J.F. (2018). Myasthenia gravis: The role of complement at the neuromuscular junction. Ann. N. Y. Acad. Sci..

[2] Sanders D.B., Wolfe G.I., Benatar M., Evoli A., Gilhus N.E., Illa I., Kuntz N., Massey J.M., Melms A., Murai H. (2016). International consensus guidance for management of myasthenia gravis. Neurology.

[3] Manu P., Rogozea L.M., Roman-Filip C. (2021). Pharmacological management of myasthenia gravis: A century of expert opinions in Cecil textbook of Medicine. Am. J. Ther..

[4] Sanders D.B., Evoli A. (2010). Immunosuppressive therapies in Myasthenia gravis. Autoimmunity.

[5] Prado M.B., Adiao K.J.B. (2023). Methotrexate in generalized myasthenia gravis: A systematic review. Acta Neurol. Belg..

[6] Bi Z., Cao Y., Liu C., Gui M., Lin J., Zhang Q., Li Y., Ji S., Bu B. (2022). Remission and relapses of myasthenia gravis on long-term tacrolimus: A retrospective cross-sectional study of a Chinese cohort. Ther. Adv. Chronic Dis..

[7] Mantegazza R., Antocci C., Peluchetti D., Sghirlanzoni A., Cornelio F. (1988). Azathioprine as a single drug or in combination with steroids in the treatment of myasthenia gravis. J. Neurol..

[8] Palace J., Newsom-Davies J., Lecky B. (1998). A randomized double-blind trial of prednisolone alone or with azathioprine in myasthenia gravis. Myasthenia gravis study group. Neurology.

[9] Myasthenia Gravis Clinical Study Group (1992). A randomized clinical trial comparing prednisone and azathioprine in myasthenia gravis. Results of the second interim analysis. J. Neurol. Neurosurg. Psychiatry.

[10] Bromberg M.B., Wald J.J., Forshew D.A., Feldman E.L., Albers J.W. (1997). Randomized trial of azathioprine or prednisone for initial immunosuppressive treatment of myasthenia gravis. J. Neurol. Sci..

[11] Rozsa C., Mikor A., Kasa K., Illes Z., Komoly S. (2009). Long-term effects of immunosuppressive treatment on myasthenia gravis. Eur. J. Neurol..

[12] Rózsa C., Mikor A., Kasa K., Illes Z., Komoly S. (2023). Mysthenia gravis treatment in the elderly presents with a significant iatrogenic risk: A multicentric retrospective study. J. Neurol..

[13] Zhang Z., Wang M., Xu L., Jiang B., Jin T., Shi T., Xu B. (2021). Cancer occurrence following azathioprine treatment in myasthenia gravis patients. A systematic review and meta-analysis. J. Clin. Neurosci..

[14] Pedersen E.G., Pottegård A., Hallas J., Friis S., Hansen K., Jensen P.E.H., Gaist D. (2014). Risk of non-melanoma skin cancer in myasthenia gravis treated with azathioprine. Eur. J. Neurol..

[15] Salort-Campana E., Laforet P., de Pouvourville G., Crochard A., Chollet G., Nevoret C., Emery C., Bouée S., Tard C. (2024). Epidemiology of myasthenia gravis in France. A retrospective claim database study (STAMINA). Rev. Neurol..

[16] Wolfe G.I., Herbelin L., Nations S.P., Foster B., Bryan W.W., Barohn R.J. (1999). Myasthenia gravis activities of daily living profile. Neurology.

[17] Crisafulli S., Boccanegra B., Carollo M., Bottani E., Mantuano P., Trifirò G., De Luca A. (2024). Myasthenia gravis treatment: From old drugs to innovative therapies with a glimpse into the future. CNS Drugs.

[18] Westerberg E., Punga A.R. (2020). Mortality rates and causes of death in Swedish myasthenia gravis patients. Neuromuscul. Disord..

[19] Basta I., PekmezoviĆ T., Peric S., NikoliĆ A., RakoČeviĆ-StojanoviĆ V., SteviĆ Z., LavrniĆ D. (2018). Survival and mortality of adult-onset myasthenia gravis in the population of Belgrade, Serbia. Muscle Nerve.

[20] Hansen J.S., Danielsen D.H., Somnier F.E., Frøslev T., Jakobsen J., Johnsen S.P., Andersen H. (2016). Mortality in myasthenia gravis. A nation-wide population-based follow-up study in Denmark. Muscle Nerve.

[21] Salahat A.A., Abdul Jabbar A.B., Sharma R., Ting Chen Y., Bernitsas E. (2025). Demographic and geographic trends in myasthenia gravis-ralated mortality in the United States, 1999–2022. Neurology.

[22] Hohlfeld R., Michels M., Heininger K., Besinger U., Toyka K.V. (1988). Azathioprine toxicity during long-term of immunosupression of generalized myasthenia gravis. Neurology.

[23] Gupta A., Goyal V., Srivastava A.K., Shukla G., Behri M. (2016). Remission and relapse of myasthenia gravis on long-term azathioprine: An ambispective study. Muscle Nerve.

[24] McGurgan I.J., McGuigan C. (2015). Non-melanoma skin cancer risk awareness in azathioprine-treated myasthenia gravis patients. Brain Behav..

[25] Kassardjian C.D., Widdifield J., Paterson J.M., Kopp A., Nagamuthu C., Barnett C., Tu K., Breiner A. (2020). Serious infections in patients with myasthenia gravis: Population-based cohort study. Eur. J. Neurol..

[26] Yaman A., Aydin F.K. (2023). Therapeutic and prognostic features in myasthenia gravis patients followed in a tertiary neuromuscular diseases center in Turkey. Front. Neurol..

[27] Zhang C., Bu B., Yang H., Wang L., Liu W., Duan R.-S., Zhang M., Zeng P., Du C., Yang L. (2020). Immunotherapy choice and maintenance for generalized myasthenagravis in China. CNS Neurosci. Ther..

[28] Fonseca V., Havard C.W. (1990). Long termtreatment of myasthenia gravis with azathioprine. Postgrad. Med. J..

[29] Narayanaswami P., Sanders D.B., Thomas L., Thibault D., Blevins J., Desai R., Krueger A., Bibeau K., Liu B., Guptill J.T. (2024). Comparative effectiveness of azathioprine and mycophenolate-mofetil for myasthenia gravis (PROMISE-MG): A prospective cohort study. Lancet Neurol..

[30] Gilhus N.E., Andersen H., Andersen L.K., Boldingh M., Laakso S., Leopoldsdottir M.O., Madsen S., Piehl F., Popperud T.H., Punga A.R. (2024). Generalized myasthenia gravis with acetylcholine receptor antibodies. A guidance for treatment. Eur. J. Neurol..

[31] Melzer N., Ruck T., Fuhr P., Gold R., Hohlfeld R., Marx A., Melms A., Tackenberg B., Schalke B., Schneider-Gold C. (2016). Clinical features, pathogenesis, and treatment of myasthenia gravis: A supplement to the guidelines of the German neurological society. J. Neurol..

